# Cognitive impairment in cerebellar lesions: a logit model based on neuropsychological testing

**DOI:** 10.1186/s40673-017-0071-9

**Published:** 2017-07-28

**Authors:** Eva Bolceková, Matej Mojzeš, Quang Van Tran, Jaromír Kukal, Svatopluk Ostrý, Petr Kulišťák, Robert Rusina

**Affiliations:** 1 Department of Neurology and Centre of Clinical Neuroscience, First Faculty of Medicine, Charles University and General University Hospital in Prague, Prague, Czech Republic; 2 Faculty of Nuclear Sciences and Physical Engineering, Czech Technical University, Prague, Czech Republic; 3 Department of Neurology, Hospital České Budějovice, a.s., České Budějovice, Czech Republic; 4 Department of Neurosurgery and Neurooncology, First Faculty of Medicine, Charles University and Military University Hospital, Prague, Czech Republic; 5 Department of Psychology, Faculty of Arts, Charles University, Prague, Czech Republic; 6 Department of Neurology, Thomayer Hospital, Prague, Czech Republic

**Keywords:** Cerebellum, Cognition disorders, Neuropsychological tests, Attention, Executive function, Logistic models

## Abstract

**Background:**

Damage to the cerebellum may lead to motor dysfunctions, but also to the neuropsychological deficits that comprise the Cerebellar Cognitive Affective Syndrome (CCAS). It can affect executive functions, attention, memory, visuospatial functions, language, and emotions. Our goal was to determine which neuropsychological tests could be effectively used to identify this syndrome during a short examination.

**Methods:**

Twenty-five patients with an isolated cerebellar lesion and 25 matched healthy controls were examined using an extensive neuropsychological battery.

**Results:**

Logistic regression models and sub-models were computed for individual tests, as well as for the full battery. The best results were produced by a model combining patient education level, the number of errors on the California Verbal Learning Test, and time on Prague Stroop Test (Dots).

**Conclusions:**

Based on the results, we suggest that a condensed battery of neuropsychological tests can be used to detect CCAS. The tests are easy to administer and could be helpful in both research and clinical settings.

**Electronic supplementary material:**

The online version of this article (doi:10.1186/s40673-017-0071-9) contains supplementary material, which is available to authorized users.

## Background

The cerebellum has long been known to influence motor skills such as posture, gait, balance, and movement coordination. However, its role in cognition and emotion was discovered relatively recently. A possible relationship between the cerebellum and higher cognitive functions, based on neurological findings, was proposed in the 1980s [1]. A decade later, Schmahmann and Sherman [2] defined a new clinical entity, Cerebellar Cognitive Affective Syndrome (CCAS), as an impairment in four areas of cognitive functioning: (1) executive functions (planning, set shifting, abstract reasoning, working memory, verbal fluency), (2) visuospatial functions (visuospatial organization and memory), (3) personality (blunting of affect, disinhibited or inappropriate behavior), and (4) language functions (dysprosodia, agrammatism, mild anomia). CCAS, or Schmahmann’s syndrome, constitutes the third key element of clinical ataxiology, after cerebellar motor syndrome and vestibulo-cerebellar syndrome [3].

The exact nature and cause of CCAS is still under study. In their very detailed meta-analysis, O’Halloran, Kinsella and Storey [4] reviewed different cognitive domains and neuropsychiatric diagnoses, in which cerebellar involvement had been shown. They conclude that while the dispute over the precise picture of CCAS continues, neuropsychological deficits were consistently shown in the areas of executive functions, attention, learning and memory, language, and visuospatial functions.

Executive functions seem to have the clearest link to the cerebellum. Published data indicate cerebellar involvement in working memory, multitasking, or response inhibition [5], as well as verbal and nonverbal fluency or concept formation [6]. The reported attention impairments include reaction times, divided attention and working memory, and both parts of the Trail Making Test [6]; and significant impairments in learning and memory, specifically in the delayed recall and visual memory, were also found [6]. Language functions influenced by the cerebellum include verbal fluency, lexical retrieval, syntax, and in some cases also reading and writing [7]. Visuospatial deficits associated with cerebellar lesions include impaired performance on line orientation tasks, mental rotations, spatial sequence processing [8], or Block Design [6].

The affective part of the CCAS was reviewed in detail by Schutter and Van Honk [9]. The authors describe the function of the cerebellum with regard to negative emotions, such as fear, anxiety and sad mood, but also its association with positive emotional states. Changes in the cerebellum were found in some neuropsychiatric diagnoses, including depression, schizophrenia, and ADHD. Typical symptoms are behavioral disinhibition, emotional instability, aggressive outbursts, or pathological laughing and crying (see [4]). In this aspect, specifically the cerebellar vermis plays an important role [10].

Despite some differences regarding specific areas, most authors in the field now agree that the cerebellum plays an important role in cognitive and emotional processing. This was further confirmed in a large study by Tedesco et al. [11], which described the cerebellar cognitive profile based on data obtained from 156 patients. Impairment of executive functions, as a prominent feature of the CCAS, can be explained by interconnections between the cerebellum and the prefrontal cortex, as demonstrated by cerebello-cerebral diaschisis [12].

Cerebellar dysfunction can influence a patient’s everyday life in many ways. Evidence of this was presented in a study using both neuropsychological tests and real-life tasks from the Multiple Errands Test [13]. The authors found that cerebellar lesions lead to impairments in everyday executive function abilities involving planning and multitasking.

Although motor deficits following cerebellar damage are often more apparent, the non-motor impairment, often underdiagnosed and underestimated, can be even more troubling. Moreover, caregivers often carry more burden than patients, since patients may not be aware of the changes.

In research studies, CCAS is often examined using an extensive neuropsychological battery, which is quite demanding on both patient and examiner. If CCAS patients could be identified more effectively, it would benefit both research and clinical practice. It would allow quicker education of patients, and their families, regarding possible difficulties as well as quicker entry into cognitive rehabilitation or neuropsychotherapy. Therefore, the focus of this study was to identify CCAS sensitive tests and incorporate them into a short battery that could be used in a clinical setting to quickly identify CCAS patients.

## Methods

The test group consisted of 25 patients with an isolated and clearly bordered lesion of the cerebellar hemisphere and/or the vermis (18 males and 7 females, age 53.8 ± 18.6 years, education 14.4 ± 3.5 years). All patients were right-handed. Twelve patients had left-sided damage, 11 had right-sided damage and two patients had bilateral damage. In 15 cases the etiology was ischemic stroke; the other 10 cases involved an isolated tumor (see Table 1). All patients were examined within 6 months following stroke or surgery. Patients with tumors underwent surgery between one and 3 weeks after the tumor was detected, and none of them received oncologic treatment at the time of assessment. We excluded patients with focal lesions of other parts of the brain, patients with ischemic white matter lesions, pre-existent cognitive impairment and dementia, seizures, drug or alcohol abuse, and depression and other psychiatric illnesses.

**Table 1:** Characteristics of subjects (demographic data, site of cerebellar lesion, volume of the lesion in cm^3^, and etiology). M – male, F – female, age and education are given in years

	Patients						Control		
N	Gender	Age	Education	Lesion site	Lesion volume	Lesion etiology	Gender	Age	Education
1	M	57	13	Left hemisphere	17.79	Tumor	M	67	13
2	F	51	18	Left hemisphere	31.92	Tumor	F	55	14
3	M	79	13	Left hemisphere	16.24	Stroke	F	79	12
4	M	57	18	Both hemispheres + vermis	87.51	Stroke	F	67	14
5	M	22	12	Left hemisphere + vermis	67.50	Tumor	F	50	18
6	F	68	10	Right hemisphere	26.52	Tumor	M	50	19
7	M	70	13	Right hemisphere	45.45	Stroke	F	75	14
8	M	47	23	Left hemisphere	15.33	Tumor	M	59	18
9	M	47	12	Left hemisphere	7.80	Stroke	M	53	18
10	F	50	13	Left hemisphere	18.14	Tumor	M	75	23
11	M	66	11	Right hemisphere	7.49	Stroke	M	57	18
12	M	26	18	Both hemispheres	1.89	Tumor	M	48	19
13	M	27	12	Right vermis	2.40	Stroke	M	29	15
14	F	79	12	Left hemisphere	10.88	Stroke	M	33	15
15	M	57	18	Right hemisphere	8.75	Stroke	M	60	17
16	F	29	15	Left hemisphere	9.92	Tumor	M	66	12
17	M	63	12	Left hemisphere	4.52	Stroke	M	70	13
18	M	50	18	Right hemisphere	2.28	Stroke	M	77	13
19	F	90	11	Left hemisphere	28.16	Stroke	F	88	12
20	F	62	13	Right hemisphere	0.56	Stroke	M	22	12
21	M	73	20	Right hemisphere	27.23	Tumor	F	29	13
22	M	68	12	Right hemisphere	49.39	Stroke	M	43	11
23	M	43	11	Right hemisphere	15.65	Stroke	M	47	12
24	M	32	13	Left hemisphere	3.89	Tumor	M	32	13
25	M	32	19	Right hemisphere	23.76	Stroke	M	32	13

The control group was composed of 25 healthy age-matched subjects with no neuropsychiatric history (18 males and 7 females, age 54.5 ± 18.2 years, education 14.8 ± 3.1 years). All control subjects were right-handed.

**Fig. 1:**
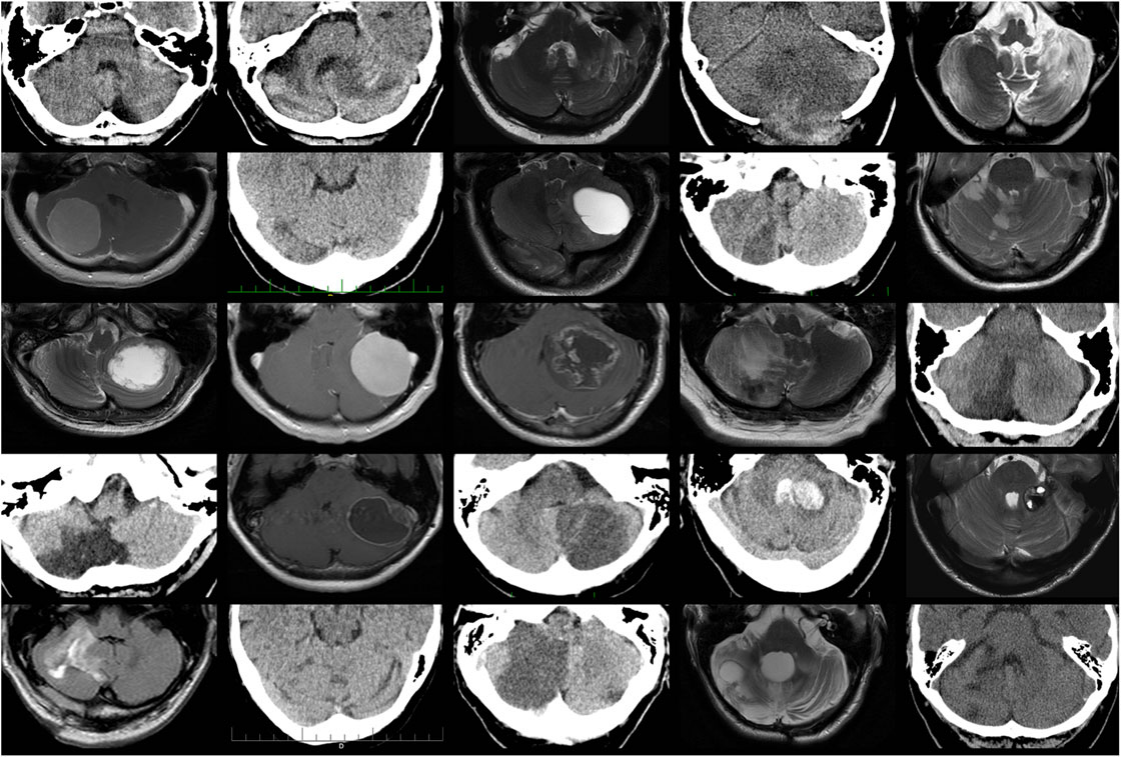
Cerebellar lesions of the patients

Members of the patient group underwent a complex neurological examination and an MRI/CT, and the lesions are illustrated in Fig. 1. This confirmed damage to the cerebellum and also confirmed that all other areas of the brain were spared. Lesion volumes were calculated by measuring three spatial dimensions and using the formula A*B*C/2, which is an approximation of the calculation of the volume of an ellipsoid, where A, B, C represent orthogonal axis lengths of the ellipsoid (axes perpendicular to each other). The exact lesion volume would be A*B*C*π/6. We approximated π/6 (= 0.52) with 0.5, which is a reasonable estimate.

All participants (test and control) were examined with a purpose-designed, complex neuropsychological battery covering a wide range of cognitive functions: attention (selective, divided, and sustained), psychomotor speed, memory (verbal, visuospatial), visuospatial functions, language, and executive functions (planning, set-shifting, verbal, and spatial fluency).

Our battery was composed of the following tests: Trail Making Test (TMT) [14], California Verbal Learning Test, 9-word version (CVLT-9) [15], Rey-Osterrieth Complex Figure (ROCF) [16], Verbal Fluency Test (VFT) [17] with Czech letters in the lexical part according to [18], Frontal Assessment Battery (FAB) [19], Five-Point Test (FPT) [20], and the Prague Stroop Test (PST) [21]. We also included a measure of emotional state, the Brunel Mood Scale (BRUMS) [22], and a scale of motor dysfunction, the International Cooperative Ataxia Rating Scale (ICARS) [23]. An overview of the methods and domains is presented in Table 2.

**Table 2:** List of neuropsychological tests used in the study

Domain	Tests used	Variables recorded
Attention, psychomotor speed	Trail Making Test (TMT)	Time in parts A and B
Verbal memory and learning	California Verbal Learning Test, 9-word version (CVLT-9)	RS – correct answers and errors (intrusions + repetitions) in trials 1–5, after interference, after 30 min delay, and in recognition
Visuospatial functions (construction and memory)	Rey-Osterrieth Complex Figure (ROCF)	RS and time in copy, recall after 3 min and after 30 min
Language	Verbal Fluency Test (VFT)	RS – correct answers for phonemic and semantic fluency in 1 min
Executive functions	Frontal Assessment Battery (FAB)	RS
	Five-Point Test (FPT)	RS – number of correct answers in 1 min
	Prague Stroop Test (PST)	Time and number of errors for Dots (D), Words (W) and Colors (C)
Emotions	Brunel Mood Scale (BRUMS)	RS – scales Tension (TEN), Anger (ANG), Depression (DEP), Fatigue (FAT), Vigor (VIG), Confusion (CON)

*RS* – raw score

The cognitive tests were chosen on the basis of our previous work, where we found significant differences between patients and healthy controls using these methods [24]. Verbal tests were completed using Czech language versions of the assessment.

The study was approved by the local ethics committee and all participants signed an informed consent.

### Statistics

Logistic regression models (logits) and their sub-models were first applied sequentially to individual psychological tests. All estimates and tests of hypotheses were performed on our group of 25 patients and 25 controls at a significance level of *p* = 0.05. Applying the maximum likelihood method to the full model, regression coefficients, their standard deviations, and *p*-values of the zero-hypothesis t-test were obtained together with the *p*-value of the likelihood ratio (LR) test of model significance. A constant logit model, with zero coefficients, was used as a reference for LR testing. Area under curve (AUC) of the best submodel was calculated. The significant explanatory variables were also described by odds ratio (OR) and its 95% CI.

All the statistical calculations were performed using the MATLAB Statistical Toolbox. The algorithm of the best sub-model selection was inspired by the work of Vahdani et al. [25], Zheng et al. [26] and Martinez et al. [27]. The selection principle and its relationship to hypothesis testing and binary optimization are described by Mojzes et al. [28].

## Results and discussion

Patient and control groups did not differ in age (W = 321.5, *p* = .9), education (W = 361, *p* = .3) or gender (χ^2^ = 0, df = 1, *p* = 1.0). The correlations between age and education and test scores were tested by Pearson correlation coefficient on critical level 0.05. In both patient and control groups, the neuropsychological test scores were influenced by age, as expected. Education did not influence any of the scores of the patients’ group (all *p* > .5), and in the control group, correlations with education were only significant for verbal fluency tests (*p* < .1). The relationship between sex and test scores was tested by Wilcoxon-Mann-Whitney rank-sum test on critical level 0.05, and no significant differences were observed.

Results of neuropsychological testing and statistical analysis (significant group differences, when tested with the non-parametric Wilcoxon-Man-Whitney test), showed significant differences between patient and control groups in performance on executive functions’ tests (FAB, TMT B, VFT, FPT, and PST), learning and memory (all measures in CVLT-9, ROCF Immediate and Delayed Recall), visuospatial functions (ROCF Copy), and attention and psychomotor speed (TMT A; see Table 3). The relationships between lesion volumes and neuropsychological scores were tested using the Pearson correlation test at the critical level of 0.05 under the supposition of a zero lesion volume in controls. The significant correlations are in Table 3. Most of the neuropsychological scores correlated significantly with lesion volume, and all the correlations were in the expected direction (i.e., larger lesions were associated with lower raw scores, longer times, and more errors).

**Table 3:** Results of neuropsychological tests

*Method*			*m* (*sd*)		WMW *p*	*Correlation with lesion volume*	*Correlation with ICARS*
			*Patients*	*Control*			
TMT	*A*	Time (s)	74.4 (53.0)	40.2 (15.5)	.003	0.70***	0.71**
	*B*	Time (s)	200.7 (135.3)	77.1 (30.7)	.005	0.51***	0.53*
	*Interference*	B/A	2.50 (1.02)	1.95 (0.51)	ns	ns	ns
CVLT-9	*Trials 1–5*	Correct	31.6 (6.82)	37.1 (5.96)	.006	−0.44**	ns
		Errors	3.92 (7.47)	0.04 (0.20)	<.0001	ns	ns
	*Short delay recall*	Correct	11.0 (4.60)	14.7 (3.25)	.003	−0.47***	ns
		Errors	1.68 (2.50)	0.44 (0.12)	.01	ns	ns
	*Long delay recall*	Correct	10.6 (4.85)	14.8 (3.46)	.002	−0.50***	ns
		Errors	1.76 (1.96)	0.36 (0.70)	.002	0.50***	ns
	*Recognition*	Correct	8.08 (1.41)	8.92 (0.28)	.003	ns	ns
		Errors	1.52 (1.90)	0.20 (0.41)	.0007	0.40**	ns
ROCF	*Copy*	RS	29.6 (7.44)	34.4 (2.12)	.009	−0.45**	ns
		Time (s)	217.7 (107.6)	157.0 (37.9)	.06	0.39**	ns
	*Short delay recall*	RS	16.7 (6.95)	20.6 (6.34)	.04	−0.45***	−0.64**
		Time (s)	147.7 (70.74)	128.5 (47.2)	ns	ns	ns
	*Long delay recall*	RS	17.0 (7.31)	20.6 (5.76)	.04	−0.42**	−0.60*
		Time (s)	98.3 (46.0)	95.9 (26.8)	ns	ns	ns
VFT	*Phonemic*	RS	30.6 (14.3)	46.4 (11.2)	.0002	−0.51***	−0.64*
	*Semantic*	RS	17.2 (7.79)	24.0 (6.85)	.01	−0.45**	ns
FPT		RS	6.84 (3.68)	8.32 (3.24)	0.06	−0.34*	−0.60*
FAB		RS	14.8 (2.71)	17.0 (1.08)	.004	−0.52***	−0.52*
PST	*Dots*	Time (s)	17.0 (5.60)	12.8 (2.81)	.0007	0.45**	ns
		Errors	0.24 (0.52)	0.00 (0.00)	.02	0.50***	0.52*
	*Words*	Time (s)	20.7 (7.49)	15.9 (4.03)	.003	0.33*	ns
		Errors	0.16 (0.62)	0.00 (0.00)	ns	ns	ns
	*Colors*	Time (s)	37.7 (23.1)	29.0 (11.4)	ns	ns	ns
		Errors	1.32 (1.52)	0.48 (0.71)	.05	0.46***	ns
	*Interference*	C/D	2.21 (1.04)	2.26 (0.71)	ns	ns	ns
BRUMS^a^	*Anger*	RS	2.68 (3.64)	1.46 (1.45)	ns	ns	ns
	*Confusion*	RS	3.91 (3.37)	1.69 (2.43)	.03	ns	ns
	*Depression*	RS	2.00 (3.09)	1.92 (2.10)	ns	ns	ns
	*Fatigue*	RS	5.14 (3.94)	4.62 (3.97)	ns	ns	ns
	*Tension*	RS	2.64 (2.61)	2.46 (2.30)	ns	ns	ns
	*Vigor*	RS	6.27 (4.42)	6.31 (2.95)	ns	−0.50*	ns

^a^ *N* = 22 patients +13 control subjects

*p < .05, *p < .01, ***p < .0001

ns = not significant

*m* – mean, *sd* – standard deviation, RS – raw score, WMW – Wilcoxon-Mann-Whitney test, TMT – Trail Making Test, CVLT-9 – California Verbal Learning Test, 9-word version, ROCF – Rey-Osterrieth Complex Figure, VFT – Verbal Fluency Test, FAB – Frontal Assessment Battery, FPT – Five-Point Test, PST – Prague Stroop Test, BRUMS – Brunel Mood Scale, ICARS – International Cooperative Ataxia Rating Scale

The measure of emotional state was only administered to a smaller group of subjects (22 patients, 13 controls). Only the results from the Confusion subscale differed significantly between the groups. In a previous study, we used also the Questionnaire Measure of Emotional Empathy [29], and a short version of the Minnesota Multiphasic Personality Inventory [30]. Neither of them revealed statistically significant differences between the groups. We may view these negative results as a consequence of impaired understanding of one’s emotional state after cerebellar damage. The patients often exhibited emotional changes, but were not aware of them, and did not report them. The higher scores on the confusion scale of BRUMS may reflect this. We suggest that informant-based questionnaires would be more adequate measures of emotional changes in CCAS.

The ICARS was applied only to the patients group. Motor deficits were generally quite mild. Mean scores and standard deviations were as follows: Posture and Gait Disturbances 5.06 (5.63), Kinetic Function 4.81 (4.13), Speech Disorders 0.69 (1.08), Oculomotor Disorders 1.50 (1.46), and Total Score 12.06 (9.68).

We studied the relationships between ICARS scores and the scores of the other tests using the Pearson correlation coefficient. The statistically significant dependencies are reported in Table 3 and Additional file 1 Table S1 as correlation coefficients and corresponding *p*-values. We found statistically significant correlations of ICARS Total score with TMT (i.e., higher ICARS score correlated with longer time on both parts), ROCF delayed scores, FAB, phonemic VF, and FPT (i.e., higher ICARS score correlated with lower scores). Higher ICARS scores were also associated with higher number of errors on the PST D. Correlations of ICARS subscores (Posture/Gait Disorders, Kinetic Function Disorders, Speech Disorders, and Oculomotor disorders) are displayed in the Additional file 1 Table S1. The highest and most significant correlations were found with the Speech Disorders subscale. This suggests that neuropsychological test results cannot be separated from motor dysfunction, and dysarthria and other speech impairments may considerably influence the scores. However, these results should be confirmed on a larger sample.

The results of separate logistic modeling are presented in Table 4, where the regression coefficients can be seen to be rarely significant. That is why we reduced the number of explanatory variables to obtain the best sub-models, which were specified to guarantee two conditions: all coefficients are significant and the *p*-value of LR test was the smallest possible. The optimal sub-models are also presented in Tab. 4 and consist of two explanatory variables at most, and significantly differ from the constant model. Of course, all individual sub-models were better than adequate full models with regard to the *p*-values on the LR test.

**Table 4:** Individual neuropsychological tests and their best sub-models

	Full model		Sub-model		
Explanatory variable	Coefficient (*sd*)	*p*-value	Coefficient (*sd*)	*p*-value	OR [95% CI]
const	13.736 (6.867)	0.0455	8.064 (3.461)	0.0198	
Gender	−1.307 (0.946)	0.1674			
Age	−0.045 (0.024)	0.0672			
Education	0.111 (0.143)	0.4364			
ROCF copy RS	−0.444 (0.199)	0.0255	−0.246 (0.103)	0.0166	0.782 [0.639; 0.957]
ROCF copy time	0.010 (0.006)	0.1042			
ROCF recall 3 RS	−0.173 (0.196)	0.3787			
ROCF recall 3 time	0.013 (0.009)	0.1525			
ROCF recall 30 RS	0.176 (0.204)	0.3902			
ROCF recall 30 time	−0.010 (0.015)	0.4786			
	LR test	0.0070	LR test	0.0011	
const	−0.984 (2.187)	0.6527	−1.877 (0.814)	0.0211	
Gender	−0.378 (0.826)	0.6475			
Age	−0.050 (0.024)	0.0392			
Education	0.044 (0.114)	0.7016			
TMT A	0.026 (0.029)	0.3613			
TMT B	0.020 (0.013)	0.1271	0.018 (0.009)	0.0332	1.018 [1.001; 1.035]
	LR test	0.0017	LR test	0.0002	
const	1.219 (12.619)	0.9230	−2.111 (0.624)	0.0007	
Gender	−0.443 (1.475)	0.7639			
Age	−0.018 (0.046)	0.6912			
Education	0.079 (0.213)	0.7104			
CVLT-9 1–5	−0.143 (0.262)	0.5855			
CVLT-9 1–5 errors	3.591 (1.943)	0.0646	3.423 (1.242)	0.0058	30.66 [2.688; 349.8]
CVLT-9 short delay	−0.552 (0.598)	0.3561			
CVLT-9 short delay errors	−1.005 (1.033)	0.3303			
CVLT-9 long delay	0.786 (0.794)	0.3224			
CVLT-9 long delay errors	0.335 (1.349)	0.8040			
CVLT-9 recognition	−0.188 (1.176)	0.8733			
CVLT-9 recognition errors	1.941 (1.073)	0.0706	1.280 (0.637)	0.0444	3.597 [1.032; 12.54]
	LR test	7.32 × 10^−6^	LR test	1.73 × 10^−9^	
const	5.282 (2.708)	0.0511	3.562 (1.151)	0.0020	
Gender	−0.945 (0.923)	0.3059			
Age	−0.037 (0.025)	0.1446			
Education	0.139 (0.128)	0.2792			
FPT	0.085 (0.143)	0.5520			
VFT - phonemic	−0.124 (0.051)	0.0139	−0.091 (0.027)	0.0009	0.913 [0.866; 0.963]
VFT - category	−0.022 (0.072)	0.7653			
	LR test	0.0025	LR test	0.0001	
const	−3.245 (5.694)	0.5687	−4.031 (1.535)	0.0086	
Gender	−0.265 (0.868)	0.7606			
Age	−0.039 (0.026)	0.1450			
Education	−0.010 (0.127)	0.9383			
PST D time	0.614 (0.515)	0.2338	0.282 (0.110)	0.0102	1.326 [1.069; 1.645]
PST D errors	24.000 (32,414.005)	0.9994			
PST W time	−0.221 (0.221)	0.3170			
PST W errors	13.982 (4072.457)	0.9973			
PST C time	−0.065 (0.197)	0.7425			
PST C errors	0.378 (0.480)	0.4311			
PST interference	0.966 (2.826)	0.7324			
	LR test	0.0185	LR test	0.0007	
const	14.198 (4.887)	0.0037	9.256 (3.368)	0.0060	
Gender	−0.101 (0.776)	0.8964			
Age	−0.041 (0.023)	0.0728			
Education	0.039 (0.109)	0.7166			
FAB	−0.774 (0.259)	0.0028	−0.572 (0.203)	0.0049	0.564 [0.379; 0.840]
	LR test	0.0022	LR test	0.0003	

Adequate parameter estimates (coefficients), their standard deviation, and *p*-values of the t-test are included. *sd* – standard deviation, OR – odds ratio, CI – confidence interval, LR – likelihood ratio, const – constant value, RS – raw score, ROCF – Rey-Osterrieth Complex Figure, TMT – Trail Making Test, CVLT-9 – California Verbal Learning Test, 9-word version, FPT – Five-Point Test, VFT – Verbal Fluency Test, PST – Prague Stroop Test, FAB – Frontal Assessment Battery

The same estimations were performed for a complete battery of tests using 30 explanatory variables in the full model. Unfortunately, the data in this case was linearly separable, which prevented statistical analysis. However, there was a best sub-model and it includes only three explanatory variables. The properties of complete condensed model are presented in Table 5. The complete condensed model has the lowest *p*-value on the LR test and therefore, it was better than any individual sub-model.

**Table 5:** The best sub-model of complete test battery

	Sub-model		
Explanatory variable	Coefficient (*sd*)	*p*-value	OR [95% CI]
const	−14.448 (5.247)	0.0059	
Education	0.397 (0.202)	0.0493	1.487 [1.001; 2.210]
CVLT-9 1–5 errors	5.821 (2.022)	0.0040	337.3 [6.410; 17,750]
PST D time	0.452 (0.155)	0.0035	1.571 [1.160; 2.129]
	LR test	6.81 × 10^−10^	

*sd* – standard deviation, OR – odds ratio, CI – confidence interval, LR – likelihood ratio, const – constant value, CVLT-9 – California Verbal Learning Test, 9-word version, PST D – Prague Stroop Test (Dots)

The leave-one-out strategy of cross-validation was used for the verification of all sub-models. This procedure consists of the application of logistic regression to all data samples except one. The excluded sample is used for the calculation of its output signal. After all exclusions, the output signals were tested using Wilcoxon-Mann-Whitney rank-sum test on critical level 0.05. The corresponding *p*-values of output differences between patients and control group were all significant and ranged from 0.01 to 0.0002. It can be seen that the outputs of all optimal sub-models significantly differentiate patients from controls.

**Fig. 2:**
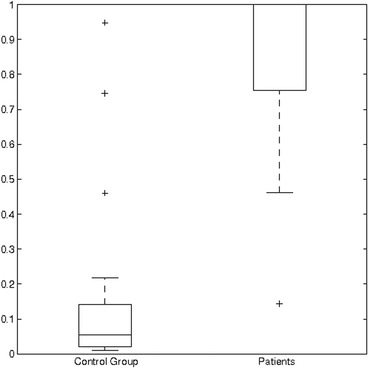
Boxplot of the distribution of control subjects and patients according to the best logit model. The value on y-axis represents the logit of the model. Ends of the whiskers represent 1.5 interquartile ranges (IQR)

Figure 2 illustrates the distribution of healthy control subjects and patients with cerebellar damage according to the best model. The groups were well separated from each other. We also performed receiver operating characteristics (ROC) analysis which resulted in an area under curve (AUC) of 0.99 for the best model.

These results suggest that the presented logit model provides very good predictive value for CCAS. The probability of CCAS would be given by the equation:

logit (y) = −14.448 + 0.397*EDU + 5.821*CVLT1-5_err +0.452*PST_D

where y is the probability of CCAS. A convenient table for computing the probability is provided as Electronic Supplementary Material.

Additionally, post-estimation analysis of model sensitivities and specificities was performed using leave-one-out cross-validation scheme for various model orders of fixed explanatory variables. The results are displayed in Table 6 as experimental sensitivities and specificities, i.e. point estimates of adequate probabilities and their corresponding 95% CI. The out of sample diagnostics of these models demonstrate their relatively limited predictive abilities. The best prediction result is obtained with a submodel with CVLT-9 predictors rather than the full model with all predictors. Since the models are statistically significant, their significance can indicate the existence of certain causality. However, they might only provide some supporting information during a diagnostic process rather than absolute predictability.

**Table 6:** Post-estimation analysis of sub-models

Model	Sensitivity [95% CI]	Specificity [95% CI]
ROCF	65.00 [58;72]	60.00 [46;74]
TMT	72.73 [60;84]	67.84 [54;80]
CVLT-9	80.05 [70;92]	72.41 [60;84]
VF	76.19 [64;88]	68.97 [56;82]
FAB	69.57 [56;82]	66.67 [54;80]
Full	75.00 [62;86]	73.08 [60;84]

CI – confidence interval, ROCF – Rey-Osterrieth Complex Figure, TMT – Trail Making Test, CVLT-9 – California Verbal Learning Test, 9-word version, VFT – Verbal Fluency Test, FAB – Frontal Assessment Battery

Similar to other cognitive impairments, there is no consensus about a single neuropsychological test battery suitable for CCAS, which has resulted in different tests being used at different research centers. Our battery was specifically designed to assess the principal domains affected in CCAS patients, executive functions, visuospatial functions and memory, personality changes, and language [2].

Cerebellar involvement in explicit memory has been shown in several studies [31–34]. We chose the CVLT-9, a test of verbal memory, for its relatively low difficulty, since it uses only nine words, as well as for its use of category cues. Cerebellar involvement in executive functions is well established [35], therefore we included more measures of this domain: FAB for a general level, TMT B for set-shifting, PST for interference, fluency tests (verbal and design) for planning, innitiation, flexibility, and perseveration. Visuospatial domain deficits in cerebellar patients were found by Molinari et al. [36]. In our study, visuoconstruction was assessed with the ROCF. For language assessment, verbal fluency tests were used. They combine language skills with an executive component and therefore are sensitive to a broad range of neurological damage. Attention and psychomotor speed are closely associated with the cerebellum [37, 38]; we used the TMT A, simple color naming on the PST, and time on the ROCF drawing test, to assess them.

Our results are in accordance with other studies in terms of cognitive deficits following cerebellar damage [11]. In an earlier study involving neuropsychological deficits in patients with cerebellar lesions, we found that changes in executive functions are the most pronounced, followed by visuospatial and construction impairment, whereas attention, learning and memory were less impaired [24].

In the present study, we attempted to identify a short battery of tests that would be sensitive to CCAS. The model suggested by logistic regression analysis is based on the subjects’ education, the number of errors (intrusions and repetitions) on the CVLT-9 and time on the PST D. Both the number of CVLT-9 errors and the time on the PST D have a statistically significant positive correlation with the probability of CCAS. Level of education was a compensatory variable for the higher scores of patients with higher levels of education.

The fact that a greater weight was given to the number of CVLT-9 errors might seem surprising. Cerebellar involvement in explicit memory, which corresponds to verbal learning tasks, has not been consistently shown [39–43], although some authors have suggested its involvement in retrieval through connections with prefrontal regions [31, 32]. In our patients, memory was clearly involved, both immediate and delayed, in both verbal (CVLT-9) and visual modalities (ROCF). Our logistic model, however, did not consider the most common measure, i.e. the number of recalled words, but instead used the number of errors. We consistently found more intrusions and repetitions in our subjects with cerebellar damage. We think that this feature is consistent with the “dysmetria of thought” hypothesis [44]. The patients are not able to restrict their answers to the correct ones and make mistakes. This concept is supported by Cabeza and St Jacques [33], who have suggested that the cerebellum is involved in generating ‘candidate responses’ during retrieval, and damage to this structure could lead to distorted responses.

The second value in our logit model is represented by time spent on simple color naming (Dots) on the PST. Cerebellar involvement in psychomotor speed is well known [37, 38, 45]. Early on, Schmahmann [46], who is credited with describing CCAS, recognized the speed component in CCAS: he noted that the cerebellum regulates speed, consistency, and appropriateness of cognitive processes. In our model, color naming time was preferred over time on the TMT A, which also requires involvement of motor cortex structures [47]. It could also be that motor impairments were more variable in our patients and verbal naming speed was a more reliable factor.

### Limitations

Our study has two main limitations. First, the relatively small sample size, which means that the groups are not necessarily representative of the population. To obtain the most valid comparison and instead of using normative test data, we enrolled healthy control subjects that were paired on the recorded demographic variables (gender, age, and education) and all were Caucasian as well as being right-handed. From a statistical point of view, the small sample size could have caused biased values in the parameters of the proposed models. Other factors could have also influenced the scores, such as the subjects’ personality and emotional state, medication, or situation variables. The patients were assessed in different hospitals and as such the conditions were not completely the same; however, a quiet, separate room was always available for the examinations.

Secondly, we only assessed patients with circumscribed lesions of the cerebellum, which might be considered a design flaw. It would be interesting to assess the results of the proposed model on more diffuse cerebellar involvement. These could include other types of cerebellar damage (e.g., degenerative disorders or cerebellitis) or other neurological conditions. Therefore, we can deem our results as a proof of concept, which now needs to be validated in a larger group.

## Conclusions

The presented data suggest that a condensed battery of neuropsychological tests can be used to detect CCAS. Such a battery would be helpful in both research and clinical settings. The battery we suggest consists of only two tests: the CVLT-9 and PST. However, further research is needed to confirm our results. The proposed methods require about 5 min to administer. We suggest administration start with CVLT-9 (5 trials) followed by the PST. After entering the results into the equation (see Results Section and Supplementary Excel file), the probability of CCAS can be estimated. In a simplified interpretation, results greater than 0.5 indicate CCAS.

Assessment of CCAS offers an opportunity to educate patients and caregivers regarding potential issues related to the patient’s efficiency of problem-solving, speed of thinking, and tendencies toward certain types of errors. Possible affective changes should also be discussed. Once CCAS has been identified, cognitive rehabilitation or neuropsychotherapy can then be recommended.

## Additional file

Additional file 1: Table S1. Statistically significant correlation of neuropsychological test scores with ICARS. Pearson correlation coefficients and corresponding *p*-values are reported. ICARS – International Cooperative Ataxia Rating Scale, TMT – Trail Making Test, CVLT-9 – California Verbal Learning Test, 9-word version, ROCF – Rey-Osterrieth Complex Figure, VFT – Verbal Fluency Test, FAB – Frontal Assessment Battery, FPT – Five-Point Test, PST – Prague Stroop Test, BRUMS – Brunel Mood Scale, RS – raw score. (DOCX 8 kb)

## Abbreviations

AUC: Area under curve
BRUMS: Brunel mood scale
CCAS: Cerebellar cognitive affective syndrome
CVLT-9: California verbal learning test, 9-word version
FAB: Frontal assessment battery
FPT: Five-point test
ICARS: International cooperative ataxia rating scale
LR: Likelihood ratio
OR: Odds ratio
PST: Prague Stroop test
ROCF: Rey-Osterrieth complex figure
RS: Raw score
TMT: Trail making test
VFT: Verbal fluency test
