# Supplementary material for: Cognitive impairment in cerebellar lesions: a logit model based on neuropsychological testing
Source: Cerebellum Ataxias. 2017 Jul 28;4:13. doi: 10.1186/s40673-017-0071-9 (PMC5534033; doi:10.1186/s40673-017-0071-9)
Supplement: Additional file 1: Table S1. — Statistically significant correlation of neuropsychological test scores with ICARS. Pearson correlation coefficients and corresponding p-values are reported. ICARS – International Cooperative Ataxia Rating Scale, TMT – Trail Making Test, CVLT-9 – California Verbal Learning Test, 9-word version, ROCF – Rey-Osterrieth Complex Figure, VFT – Verbal Fluency Test, FAB – Frontal Assessment Battery, FPT – Five-Point Test, PST – Prague Stroop Test, BRUMS – Brunel Mood Scale, RS – raw score. (DOCX 8 kb) [file 40673_2017_71_MOESM1_ESM.docx]

**Supplementary table:** Statistically significant correlation of neuropsychological test scores with ICARS. Pearson correlation coefficients and corresponding *p*-values are reported.

ICARS – International Cooperative Ataxia Rating Scale, TMT – Trail Making Test, CVLT-9 – California Verbal Learning Test (9 words), ROCF – Rey-Osterrieth Complex Figure, VFT – Verbal Fluency Test, FAB – Frontal Assessment Battery, FPT – Five Points Test, PST – Prague Stroop Test, BRUMS – Brunel Mood Scale, RS – raw score.

|  | | | ICARS | | | | | |
| --- | --- | --- | --- | --- | --- | --- | --- | --- |
|  |  |  | Posture and Gait | Kinetic Function | Speech Disorders | Oculomotor Disorders | Total Score | |
| Gender | | | *ns* | *ns* | *ns* | *ns* | *ns* | |
| Age | | | *ns* | *ns* | *ns* | *ns* | *ns* | |
| Education | | | *ns* | *ns* | *ns* | *ns* | *ns* | |
| TMT | *A* | Time (s) | 0.736** | *ns* | *ns* | *ns* | 0.712** | |
|  | *B* | Time (s) | 0.514* | *ns* | 0.593* | *ns* | 0.529* | |
|  | *Interf.* | B/A | *ns* | *ns* | *ns* | *ns* | *ns* | |
| CVLT-9 | *Trials 1-5* | Correct | *ns* | *ns* | *ns* | *ns* | *ns* | |
|  |  | Errors | *ns* | *ns* | 0.729** | *ns* | *ns* | |
|  | *Short delay* | Correct | -0.530* | *ns* | -0.570* | *ns* | *ns* | |
|  |  | Errors | *ns* | *ns* | 0.880*** | *ns* | *ns* | |
|  | *Long delay* | Correct | -0.592* | *ns* | -0.603* | *ns* | *ns* | |
|  |  | Errors | *ns* | *ns* | 0.699** | *ns* | *ns* | |
|  | *Recognition* | Correct | *ns* | *ns* | *ns* | *ns* | *ns* | |
|  |  | Errors | *ns* | *ns* | 0.618* | *ns* | *ns* | |
| ROCF | *Copy* | RS | *ns* | *ns* | *ns* | *ns* | *ns* | |
|  |  | Time (s) | *ns* | *ns* | 0.553* | *ns* | *ns* | |
|  | *Short delay* | RS | -0.581* | *ns* | *ns* | *ns* | -0.644** | |
|  |  | Time (s) | *ns* | *ns* | *ns* | *ns* | *ns* | |
|  | *Long delay* | RS | -0.572* | *ns* | *ns* | *ns* | -0.600* | |
|  |  | Time (s) | *ns* | *ns* | *ns* | *ns* | *ns* | |
| VFT | *Phonemic* | RS | -0.624* | *ns* | -0.641* | *ns* | -0.638* | |
|  | *Semantic* | RS | *ns* | *ns* | -0.740** | *ns* | *ns* | |
| FPT | | RS | *ns* | -0.562* | *ns* | *ns* | | -0.602* |
| FAB | | RS | *ns* | *ns* | -0.516* | *ns* | | -0.515* |
| PST | *Dots* | Time (s) | *ns* | *ns* | *ns* | *ns* | *ns* | |
|  |  | Errors | *ns* | *ns* | *ns* | *ns* | 0.515* | |
|  | *Words* | Time (s) | *ns* | *ns* | *ns* | *ns* | *ns* | |
|  |  | Errors | *ns* | *ns* | *ns* | *ns* | *ns* | |
|  | *Colors* | Time (s) | *ns* | *ns* | 0.674** | *ns* | *ns* | |
|  |  | Errors | 0.544* | *ns* | 0.669** | *ns* | *ns* | |
|  | *Interf.* | C/D | *ns* | *ns* | 0.575* | -0.516* | *ns* | |

* *p* < .05, * *p* < .01, *** *p* < .0001, *ns* = not significant.

The ICARS Posture and Gait Disorders subscale scores correlated positively with both parts of TMT (but not the interference score), and negatively with phonemic VF. It is not clear, why Posture and Gait should correlate with these scores, and probably a more general impairment should be seen as an explanantion. The Kinetic Disorders subscale correlated negatively with FPT score. This could be explained by the fine motor functions covered by this subscale. However, other tasks that required visuoconstruction skills (TMT, ROCF) did not correlate with it. The Speech Disorders subscale correlated positively with ROCF copy time, TMT B, and time in PST C; and negatively with both verbal fluencies. It is interesting that ROCF correlates with a speech scale, even if this is clearly a visuoconstruction task. Also TMT B, which requires mainly executive funkctions, seems to be influenced by it. The verbal fluency is quite understandably connected to this scale (the two items in this subscale assess verbal fluency and dysarthria). Also speed in PST C requires fast speech. However, other two conditions of PST did not correlate with this scale, and therefore it appears that the component that is affected may be more executive (interference).
